# Impaired Dendritic Cell Homing in COVID-19

**DOI:** 10.3389/fmed.2021.761372

**Published:** 2021-11-04

**Authors:** Lukas Borcherding, Alime Sema Teksen, Bianca Grosser, Tina Schaller, Klaus Hirschbühl, Rainer Claus, Oliver Spring, Michael Wittmann, Christoph Römmele, Éva Sipos, Bruno Märkl

**Affiliations:** ^1^General Pathology and Molecular Diagnostics, Medical Faculty, University of Augsburg, Augsburg, Germany; ^2^Visiopharm, Hørsholm, Denmark; ^3^Hematology and Oncology, Medical Faculty, University of Augsburg, Augsburg, Germany; ^4^Anesthesiology and Operative Intensive Care Medicine, Medical Faculty, University of Augsburg, Augsburg, Germany; ^5^Internal Medicine III–Gastroenterology, University Hospital of Augsburg, Augsburg, Germany

**Keywords:** dendritic cells, maturation, homing, SARS-CoV-2, COVID-19, diffuse alveolar damage (DAD), multiplexed immunofluorescence, artificial intelligence

## Abstract

The high mortality of COVID-19 is mostly attributed to acute respiratory distress syndrome (ARDS), whose histopathological correlate is diffuse alveolar damage (DAD). Furthermore, severe COVID-19 is often accompanied by a cytokine storm and a disrupted response of the adaptive immune system. Studies aiming to depict this dysregulation have mostly investigated the peripheral cell count as well as the functionality of immune cells. We investigated the impact of SARS-CoV-2 on antigen-presenting cells using multiplexed immunofluorescence. Similar to MERS-CoV and SARS-CoV, SARS-CoV-2 appears to be impairing the maturation of dendritic cells (DCs). DC maturation involves a switch in surface antigen expression, which enables the cells' homing to lymph nodes and the subsequent activation of T-cells. As quantitative descriptions of the local inflammatory infiltrate are still scarce, we compared the cell population of professional antigen-presenting cells (APC) in the lungs of COVID-19 autopsy cases in different stages of DAD. We found an increased count of myeloid dendritic cells (mDCs) in later stages. Interestingly, mDCs also showed no significant upregulation of maturation markers in DAD-specimens with high viral load. Accumulation of immature mDCs, which are unable to home to lymph nodes, ultimately results in an inadequate T-cell response.

## Introduction

Respiratory failure/acute respiratory distress syndrome (ARDS) has been identified as the leading cause of death in COVID-19 by numerous studies (1–4). Diffuse alveolar damage (DAD) is the usual histopathological correlate of COVID-associated ARDS (5–10). Complications contributing to organ failure and death in severe cases of COVID-19 include a dysregulated immune system accompanied by a cytokine storm (11, 12). Previous attempts to substantiate this dysregulation mostly investigated the peripheral blood cell count or performed single-cell analyses, while some have used immunohistochemistry or immunofluorescence to depict the inflammatory infiltrate (9, 13–15).

The role of dendritic cells (DCs) in COVID-19 has only recently begun to attract the attention of researchers. DC numbers in bronchoalveolar lavage fluids (BALF) decrease in later stages of COVID-19 (16). The term “dendritic cell” is used to describe functionally similar cells of different origins, both capable of activating T cells and inducing an adaptive immune response. CD11c^−^ plasmacytoid dendritic cells (pDCs) derive from a lymphoid precursor, whereas CD11c^+^ myeloid/conventional dendritic cells (mDCs/cDCs) are closely related to monocytes and derive from a myeloid progenitor cell. (17) Dendritic cells can be thought of as a link between the innate and the adaptive immune system (18). While they usually only make up for a small part of immune cells in lungs (19), their number rapidly increases shortly after the onset of an inflammatory process. In most cases, a normalization of the DC count can be observed after resolving the inflammation, although there are some reports of a hampered restitution of the count in some viral inflammations (17). In COVID-19, there seems to be a decline in the count of pDCs (20) and a shift toward mDCs (21, 22) in the peripheral blood.

To our knowledge no attempt has been made yet to correlate the inflammatory infiltrate with various stages of diffuse alveolar damage. Here, we quantified professional antigen-presenting cells in all stages of COVID-19 associated DAD using multiplexed immunofluorescence staining.

## Materials and Methods

### Cohort and Samples

Lung tissue was acquired from all COVID-19 associated autopsy cases of the University Medical Center of Augsburg, Germany, during the local first wave of the pandemic in 2020 (*n* = 19, Table 1). All patients had been tested positive for SARS-CoV-2. Post-mortem SARS-CoV-2 RNA was detectable in swabs of the upper and lower airways (Pharynx, Trachea, and Bronchus) at the time of autopsy in all but two cases *via* RT-qPCR (S-gene, N-gene, ORF1-gene). Respiratory insufficiency was the determining cause of death in 12 cases, while all but one of the remaining causes (septic shock) were COVID-associated, too. One patient had died of cardiac failure, showing only limited DAD patterns in the lungs.

**Table 1:** Overview over demographics, comorbidities, and systemic treatment of all 17 patients included in the final analysis.

**Demographics**	
Age (mean/range)	73 (57–90)
BMI > 25	13 (77%)
BMI (mean/range)	33.4 (19.6–66.2)
Sex (f/m)	4/13
Hospitalization in days (mean/range)	15.9 (1–36)
**Comorbidities**	
COPD	2 (12%)
Diabetes	5 (29%)
Chronic kidney disease	
Arteriosclerosis	8 (47%)
Hyperlipoproteinemia	4 (24%)
Hypertension	12 (71%)
History of malignant disorder	3 (18%)
**Treatment**	
ICU treatment	13 (77%)
Invasive ventilation	8 (47%)
NI ventilation	4 (24%)
ECMO	0
Renal replacement therapy	5 (29%)
I.v. antibiotics	15 (88%)
Dexamethasone	0
Full dose anticoagulation	9 (53%)
Reconvalescent Plasma	3 (18%)
Remdesivir	1 (6%)

Respecting the wish of their next of kin, a minimal or full-body autopsy was performed, resulting in a variable number of investigated lung lobes per patient. Out of each available lung lobe, three samples were taken, formalin-fixated and paraffin-embedded. This study was approved by the internal review board of the UKA (BKF No. 2020–18) and the ethics committee of the University of Munich (Project number 20–426, COVID-19 registry of the UKA). Informed consent was collected from the next of kin for all included patients.

### Histology and Immunohistochemistry

#### Evaluation of DAD-Stages

HE-stained slides of all 249 samples were evaluated by two experienced, board-certified pathologists (TS and BM) who were blinded to the demographic and case-related data.

The presence of hyaline membranes as well as thickened alveolar walls were the required criteria for assigning a lung lobe to the acute stage of DAD (referred to as stage 1 in this paper). Samples with additional fibroblast proliferation were classified as proliferative DAD (or stage 2). Samples presenting with collagenous fibrosis were assigned the fibrotic phase (or stage 3). Results of these analyses, among others, have been published recently (23).

#### Conventional Immunohistochemistry of the Lungs

**Figure 1:**
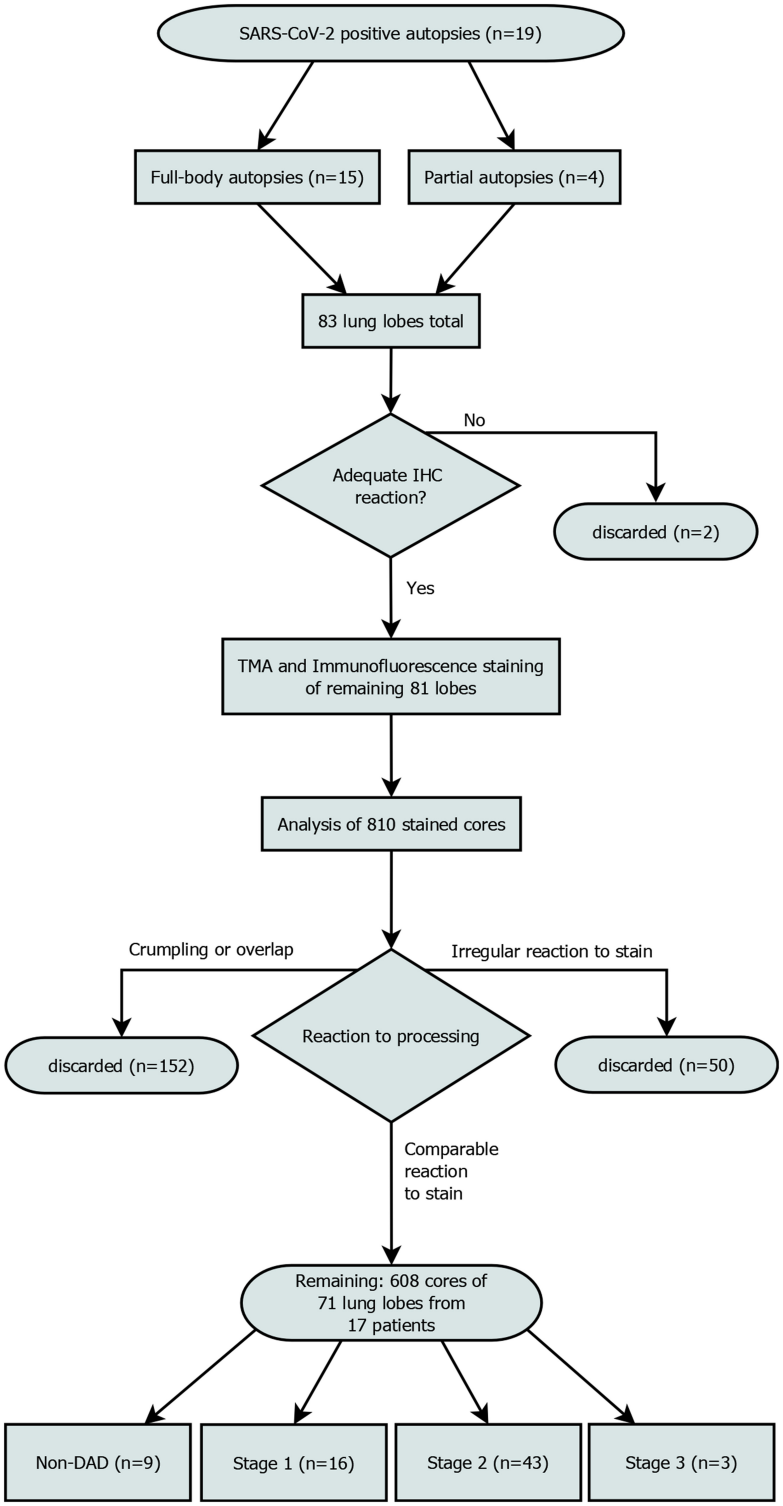
Patient enrollment flowchart. Out of 810 planned cores, 608 were included in the final analysis. “Irregular reaction to stain” refers to cores from a single patient, that showed a reaction to the stain that was not comparable to the rest of the cores. All samples of this patient were discarded.

The most properly conserved sample of each lobe was determined and subjected to further immune cell analyses. These 83 selected samples were stained with conventional immunohistochemical markers for CD3 (Clone 2GV6, 1:250, Ventana, Mannheim, Germany) and CD8 (Clone CD8/144B, ready-to-use, Cell Marque, CA, Rocklin, USA), respectively using a Leica BOND RX (Leica, Wetzlar, Germany) to evaluate the general responsiveness to antibody-staining. Both antibodies were detected by horseradish-peroxidase induced oxidation of DAB. Slides showing a satisfactory response were deemed suitable for the multiplexed immunofluorescence staining. Samples from one patient exhibited significant autolysis, reducing the number of available lung lobes from 83 to 81 and eliminating the patient from further analysis. Independently of and prior to this study the DAD-stage of each lobe had been assessed by two experienced pathologists (Figure 1).

Tissue micro-arrayed slides were further stained in conventional immunohistochemistry with markers for CD80 (ab254579Rb pAb, 1:800, abcam, Cambridge, UK) and CD86 (ab269587 Rb mAb, 1:50, abcam, Cambridge, UK), using the aforementioned DAB detection system. A double stain on CD11c (clone 5D11, 1:2 ready-to-use, Cell Marque, CA, Rocklin, USA) and CD83 (1:100, PA5-83558, ThermoFisher, Waltham, Massachusetts, USA) was performed, with DAB for CD11c and Fast Red for CD83.

#### Conventional Immunohistochemistry of Lymph Nodes

In an attempt to examine our results we stained pulmonary lymph nodes of the autopsy cases with the aforementioned CD11c antibody. We were able to assess 13 lymph nodes from 9 patients, 4 mediastinal, and 9 hilum lymph nodes. The lymph nodes were divided in three groups. Group 1 drained lung lobes of a mean DAD-stage below 1, group 2 drained lobes of a mean stage below 2, group 3 drained lobes of a mean stage below 3. As controls, in all 2021 autopsy cases, we looked for lymph nodes in cases with non-neoplastic and non-inflammatory causes of death. Three cases matched the criteria, out of which one had to be discarded due to poor fixation.

### Tissue Microarray (TMA) Construction

On the basis of whole slide scans (Pannoramic scan II, 3D Histech, Budapest, Hungary) and by using a fully automated system (TMA Grand Master by 3D Histech, Budapest, Hungary), we prepared tissue microarrayed blocks containing 10 cores of each lung (core diameter: 1.5 mm), with a maximum of 85 cores per block.

TMA of COVID-lymph nodes (diameter 1.5 mm) were constructed manually, using a Beecher Manual Tissue Arrayer (Model MTA-1, Estigen OÜ, Tartu, Estonia).

### Staining

An HE- and a multiplexed immunofluorescence-stain were performed on one slide each of a microarrayed paraffin block. For immunofluorescence staining, we used a kit with antibodies against CD20, CD68/CD168, CD11c, and MHC Class II (UltiMapper I/O APC-Kit, Ultivue, Cambridge, MA, USA) according to the manufacturer's protocol.

### Image Processing

After staining, all slides from the TMA-blocks were scanned (Pannoramic Scan II scanner, 3D-Histech, Budapest, Hungary). Scans were obtained in 20x magnification, with automated calculation of the exposure time for each of the channels (DAPI, FITC, TRITC, Cy5, Cy7) of the multiplexed immunofluorescence stain. For further analyses of the virtual slides, we used the Multiplex Phenotyping Module by Visiopharm (Visiopharm, Hørsholm, Denmark).

We discarded all cores that had significant overlap with the neighboring cores (which rendered interpretation impossible) due to processing issues, got crumpled or were not stained appropriately. Areas with lots of cell debris and strong FITC-noise interfering with the analysis were excluded from the analysis (Figure 1). This reduced the core sample count to 71 and the number of patients included to 17.

### Artificial Intelligence (AI) Analysis

The Multiplex Phenotyping Module by Visiopharm (Visiopharm, Hørsholm, Denmark), an AI-based software was trained to identify and count the different cells by surface antigen expression.

This approach offers the opportunity of automatically evaluating the cell count, not on a systemic, but a local level. It furthermore erases the need for complicated cell extraction and resulting artifacts, a problem dendritic cells are particularly prone to (17).

**Figure 2:**
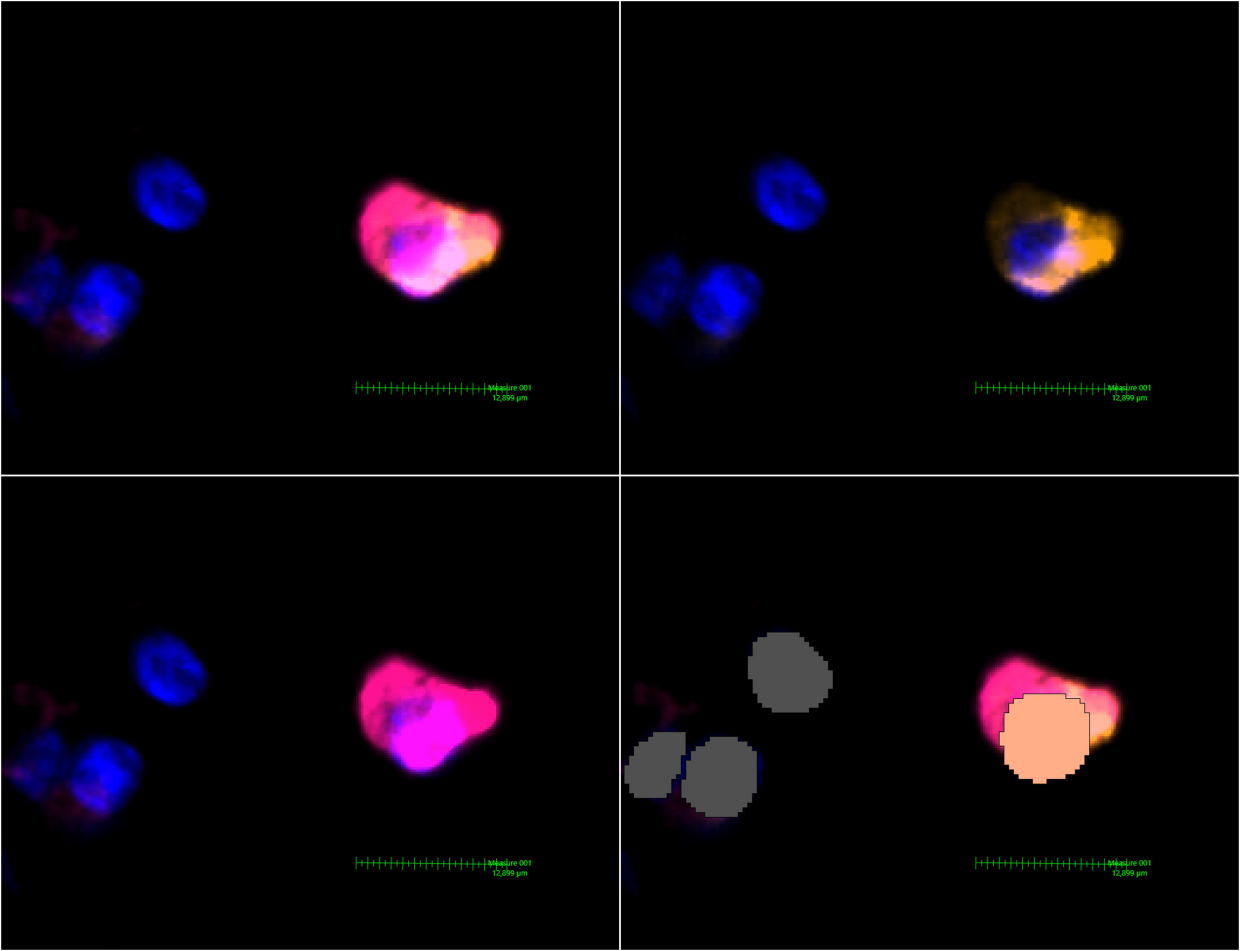
MHC class II expressing mDC found in a non-DAD lung lobe. Upper left image: Activated Channels: DAPI, Cy5 (orange, CD11c stain) and Cy7 (MHC class II stain). The comparatively weak CD11c signal can be seen alone in the upper right image. The lower left image shows Cy7 and proves a massive upregulation of MHC class II in this particular dendritic cell. The lower right image shows the interpretation by Visiopharm AI. A salmon pink mask marks a CD11c+ MHC II+ cell. Grey: Other cells (negative or below a certain threshold for markers but with a detectable nucleus in the DAPI channel).

A pre-trained, deep-learning based Nuclei Detection APP by Visiopharm (detecting DAPI-signals) was trained on a representative selection of the slides (~189,000 iterations) and then used to label and count all nuclei of each lung core (Figure 2). These labels provide the basis for further analysis by the Multiplex phenotyping module. The Phenotyping Module automatically categorizes the cells according to their antigen expression.

### IHC Analysis

Single-stain (CD80 and CD86) and double-stain (CD11c + CD83) were digitalized by a 3D Histech PANNORAMIC 1000 scanner. The resulting .mrxs-files were viewed with 3D Histech's own Case Viewer software. The double stain slides were viewed unaltered and counted manually. CD80 and CD86-stained slides were viewed using the “Gradient map visualization” plugin, which creates a heat map based on brown color signals in a digital slide and was developed for conventional IHC specifically (24). Cells surrounded by at least 50% intense positive signaling were counted as positive. For CD80, the gamma-value of all digital slides was set to 1.8 to suppress background and weak stain signals. Manual counts by LB were afterwards evaluated by a board-certified pathologist (BM). BM recounted a sample of roughly 10% of all manually counted slides. The count results were fit in a simple linear regression model [*R*^2^ = 0.9019 (double stain); *R*^2^ = 0.8228 (CD80 and CD86 count)]. CD11c stains of the lymph nodes were estimated by LS and BM.

### Statistical Analysis

The remaining 610 cores were assessed individually, whereas data of one lung lobe was compiled afterwards and statistically analyzed as a unit. To ensure comparability of lung lobes with a differing number of conserved cores, the cell count of the observed cell phenotypes in one lobe was interpreted in relation to the total number of nuclei in the lobe (for example “Macrophage Fraction” refers to the macrophage count relative to the total number of counted nuclei in the same lobe).

As cell counts showed no normal distribution, a Kruskal-Wallis test was performed on each of the mentioned cell counts to detect significant differences of the median for stages 0–2. The findings of stage 3 cores were not included for statistical testing due to the low sample size of only three remaining lobes. A Mann-Whitney-U test was used afterwards to specifically detect which two of the groups differed significantly. A *p* < 0.05 was considered significant.

We fit a simple linear regression model to correlate viral load and mDC maturation using GraphPad Prism. Data of viral load (Ct values of each lobe) were taken from the work by Hirschbühl et al. (23) and are available in the Supplementary Material of this study.

## Results

### DAD-Classification

Out of 71 lung lobes from a total of 17 patients, nine were classified as non-DAD, 16 as stage 1, 43 as stage 2 and 3 as stage 3 DAD (for an overview of the selection process see Figure 1). Five patients showed the same stage in all analyzed lobes, two of which were full body autopsies. Two cases presented with lobes that showed more than two different stages.

### Conventional Immunohistochemistry of the Lung

#### CD3 and CD8

All but two slides showed a sufficient reaction to the CD3 and CD8 stain, with abundant CD3 infiltrates and a rather scarce and dispersed reaction to CD8.

#### CD80

**Figure 3:**
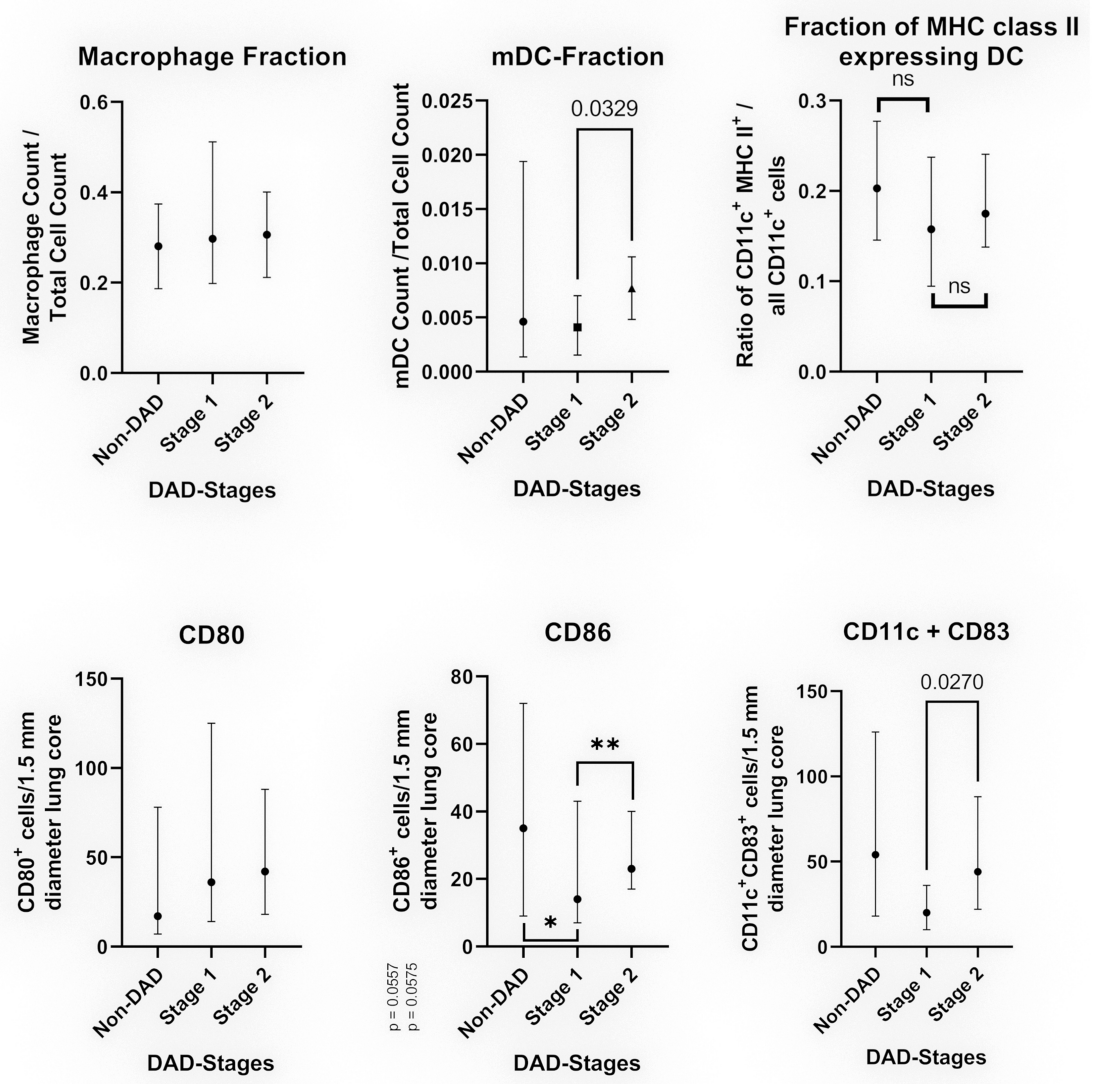
Cell populations in relation to the DAD-Stages (median with 95% CI). The top images show the results of multiplexed immunofluorescence, while the bottom images show the results of conventional IHC. Top left: Fraction CD68/CD163 positive cells in relation to the total cell count. Top middle: Fraction of myeloid dendritic cells in relation to all counted nuclei. Top right: Fraction of myeloid dendritic cells expressing MHC class II in relation to all CD11c+ cells. Bottom left: Average of CD80^+^ cells per 1.5 mm diameter lung core. Bottom middle: Average of CD86^+^ cells per 1.5 mm diameter lung core. The differences were non-significant for non-DAD vs. stage 1 (*p = 0.0557) and stage 1 vs. stage 2 (**p = 0.0575) by a narrow margin. Bottom right: Average of CD11c^+^CD83^+^ cells (double staining) per 1.5 mm diameter lung core.

The median count of CD80^+^ cells showed no statistically significant differences between non-DAD and stage 1 lobes or stage 1 and 2. The difference between non-DAD and stage 2 lobes showed a statistical significance (*p* = 0.046, Figure 3).

#### CD86

The count of CD86^+^ cells varied to a greater extend, showing a sharp decline in stage 1 compared to non-DAD and an increased count in stage 2. Both differences were non-significant by a narrow margin (*p* = 0.0557 and *p* = 0.0575, Figure 3).

#### CD11c + CD86 Double Stain

Resembling a similar pattern as the CD86 count, the count of CD11c^+^CD86^+^ cells was lower in stage 1 compared to non-DAD, with a subsequent increase in stage 2. The latter was statistically significant (*p* = 0.027).

### Conventional Immunohistochemistry of Lymph Nodes

In conventional IHC we found a rather constant CD11c^+^ cell count throughout groups 2 and 3 (with mDCs making up roughly 1/3 of all cells). Only two lymph nodes could be assigned to group 1. Both of these showed a higher CD11c count (around 2/3 of all cells). Both control lymph nodes showed roughly the same distribution as groups 2 and 3. Statistical analysis was not attempted due to the low case number.

### Multiplexed Immunohistochemistry–APC Kit

#### CD20

No statistically significant difference was detected in the CD20-Fraction (~*p* = 0.2014). While B-lymphocytes accounted only for a very small number of the cell population, three cores showed larger accumulations of CD20^+^ cells, possibly representing germinal centers. Two of these germinal centers were found in non-DAD lobes and one in a stage 2 sample.

#### CD68/163

Cells positive for CD68 or CD163 were counted as macrophages. All stages as well as non-DAD lungs showed massive macrophage infiltration. Macrophages represented a large portion of all investigated cells, with no significant differences between the stages (*p* = 0.7939) (Figure 3).

A substantial portion of macrophages expressed MHC class II proteins. The differences were not statistically significant (*p* = 0.1952, data not shown).

#### CD11c

Five outliers were detected (four in stage 2 and one in non-DAD) and subsequently removed. Two of the outliers showed an overall weak CD68/163 signal (and in turn an unusually low macrophage count), which might have led to a misclassification of macrophages as mDC.

We detected an increase in the fraction of dendritic cells (CD11c^+^, or CD11c^+^MHC II^+^) from non-DAD toward later stages. This increase was not significant when comparing non-DAD to stage 1 (*p* = 0.4896) or stage 2 (*p* = 0.4605) lung lobes. There was a statistically significant (*p* = 0.0329) raise in the dendritic cell fraction from stage 1 to stage 2 (Figure 3). CD11c^+^ cells made up 0.44% of all counted cells in stage 1 and 0.88% in stage 2. Likewise, the count of mDC in stage 3 lobes showed a non-significant increase in comparison to stage 2 samples.

No significant difference was found in the ratio of CD11c^+^MHC II^+^/all CD11c positive cells (Figure 3).

## Discussion

In this retrospective study, we performed an analysis of the immune cells involved in SARS-CoV-2 induced DAD based on 19 cases of the Augsburg autopsy series (23) using a multiplexed immunohistochemistry approach. We found an increased number of dendritic cells accumulating in later stages of DAD. This is most likely attributable to a combination of an impaired upregulation of maturation markers and a subsequent failure of homing to lymph nodes. Ultimately, an effective T cell response is prevented.

### Methodology

The UltiMapper kits rely on antibodies marked with a DNA sequence unique for every antibody-type. Signal amplification is achieved by *in-situ* amplification, reducing the risk of background noise compared to Tyramide Signal Amplification-based methods (25). This allows for the simultaneous use of up to eight different antibodies. Analyzing the generated multidimensional dataset (considering the simultaneous use of four different antibodies can indicate 2^4^ = 16 different states for a single cell) makes AI-based analysis almost mandatory. Visiopharm software has already been used in peer-reviewed studies for analyzing conventional immunohistochemistry and multiplexed immunofluorescence (26, 27).

### Dendritic Cell Migration and Maturation

Depending on their maturation state, dendritic cells express a variety of surface antigens and receptors. Chemokines like CCL3 and CCL5 are produced by a wide variety of pulmonary cells, including epithelial cells, macrophages, neutrophils and fibroblasts (28–32). These chemokines direct circulating DCs expressing CCR1 and CCR5 toward the lung (17, 33), whereas a downregulation in CCR1 and CCR5 results in impaired migration toward the site of inflammation (34). Numerous studies have shown CCR1 and CCR5-expressing DCs to migrate toward a higher CCL5-concentration (28, 35, 36), *in vitro* as well as specifically in airway mucosa (37). DCs in lungs have been shown to express both of these receptors (17). Carrying a sentinel-like function, these local immature DCs take up and process antigens. It is only in combination with danger-signals like pathogen-associated molecular patterns (PAMP) that DCs start to undergo a maturation process. At the end of this process, the matured DC has migrated to the T-cell region of lymphoid organs and gained the ability to activate naïve T-cells (a process also known as “licensing”). DCs are the most potent initiators of T-cell response (18). Maturation is accompanied by a radical shift in surface antigen expression. As mature DCs are first and foremost professional antigen-presenting cells, receptors for antigen presentation like MHC class I and II are upregulated along with co-stimulating molecules like CD40, CD80, CD83, CD86 (38–40). Chemokine receptors CCR1 and CCR5 are downregulated, ending the stationing of the cell–a process. Sozzani et al. (41) described as “weighing the anchor”. CCR7 on the other hand is upregulated and aids in recruiting the cell toward lymphatic tissue (17, 42–44). Thus, an impaired maturation process hampers DC homing, antigen presentation and ultimately impedes an effective T-cell response.

### Immune Evasion Strategies

Some immune evasion strategies of viral pathogens, including coronaviridae, aim at the maturation of DCs. MHC class I and II upregulation is impaired in MERS-CoV infected DCs (45). SARS-CoV, while able to infect monocyte-derived DCs (46), also hampered maturation of DCs (no significant upregulation of MHC class I, II, CD83, CD86) (47). Simultaneously, SARS-CoV induced the expression of CCR-1 and CCR-5 (48). For SARS-CoV-2, data so far are also pointing toward impaired maturation of mDCs (21, 22). It should be noted that while Law et al. reported a significant increase in the expression of CCR1 and CCR5, they interpreted this finding as facilitating migration from the site of infection toward lymph nodes. To our knowledge, this does not concur with current findings regarding these receptors (28, 35).

Interestingly, we found mDCs in stage 1 lung lobes also had failed to upregulate MHC class II. This is noteworthy as an inverse correlation between DAD stage and viral load has been described previously (23). Based on the data of this study, we attempted to fit a simple linear regression between the viral load and mDC maturation. A correlation could not be detected (*R*^2^ = 0.003928, *p* = 0.6256).

To confirm our findings, we attempted conventional IHC staining on other DC maturation markers, namely CD80, CD86, and CD83. The results of the CD80 count were overall non-conclusive, although there was a detectable difference between non-DAD and stage 2 lobes. It is likely that the results were skewed by the overall high count of alveolar macrophages, most of which appeared to be CD80^+^. CD80 has been shown to be expressed by macrophages (49).

As the CD83 antibody proved to be rather unspecific, we conducted a duplex stain in combination with CD11c. The distribution of double positive cells as well as of CD86^+^ cells resembled that of MHC II^+^ mDCs, with no detectable increase of activated DC in stage 1. Even more so, stage 1 lobes showed a significantly lower count than stage 2 lobes in the double stain [and a near significant difference to non-DAD as well as stage 2 in CD83 (Figure 3)].

Considering the fairly low number of available lymph nodes, especially in group one (including two cases only), the results should be interpreted very cautiously. Assuming the higher CD11c^+^ count of the two cases of group 1 is representative and does not present outliers, one could argue that this indicates an increased DC homing in very early COVID-lungs (hence a physiological response to a pathogen). In some cases though, viral immune evasion might lead to an ineffective immune response (partially caused by impaired DC homing) and to development of DAD, represented by groups 2 and 3. It should be noted that group 2 also drained lung lobes in stage 1. This interpretation is, however, highly speculative.

### Hypothesis

The simultaneous induction of receptors tying the DCs to inflammatory foci and subsequent impaired maturation are possible explanations for our findings. While immature dendritic cells keep getting recruited, they ultimately fail to fulfil their primary goal: homing to lymph nodes and activating T-lymphocytes. This results in an observable accumulation of dendritic cells in the lungs and prevents an effective T-cell response.

One recent study by Onodi et al. reports a SARS-CoV-2 induced significant upregulation of maturation markers in (CD11c^−^) plasmacytoid predendritic cells specifically (50), a subtype of DCs that is similar to mDCs regarding some functions, but of lymphoid origin. Our study, by design, only reports on CD11c^+^ mDCs. Considering the different changes of both pDCs and mDCs to COVID-19 in cell count alone, it is reasonable to assume that the molecular response could also vary to a greater extend. Further research needs to be conducted to evaluate the similarities or differences in the molecular reaction of both DC types.

It is not unlikely that severe DAD might cause an increased release of chemokines compared to earlier stages, thus causing an intensified recruitment of DCs to foci of advanced lung damage. It has indeed been established that the cytokine storm in severely affected patients explicitly implies elevated levels of CCL3 amongst other cytokines (51). Conclusive data discussing a possible correlation between cytokine levels and DAD stage specifically have not yet been published.

Furthermore, we did not detect an increased number of macrophages in later stages. Monocytes are closely related to mDCs and are known to rely on a similar pattern chemotactic factors for trafficking toward inflammation (52). This renders an elevated chemokine level in later stages even less likely to be causal for the increased local mDC count.

There are no approved drugs to specifically induce DCs maturation. Recent studies are investigating the possible use of CCR5-inhibitors in COVID-19, primarily targeting monocyte trafficking (53, 54). Future works might uncover possible effects on mDCs.

### Limitations

There are some limitations to this study.

Post-mortem investigations are prone to autolysis, possibly affecting the results. However, as insightful as *in vivo* investigations might be, lung biopsies from COVID-19 patients with ARDS are not justifiable.

Although our study included only 17 cases and should thus be interpreted with caution, these cases represent roughly 77% of all COVID-19 deceased of the first wave at the UKA. Unfortunately, we were not able to acquire a sufficient number of lymph nodes for a conclusive analysis, which might have further elucidated our hypothesis.

Lastly, CD80, CD83, and CD86 show varying specificity for activated dendritic cells, impeding IHC analysis. More detailed histological single-cell characterization could be made possible by further development of multiplexed immunohistochemistry in the foreseeable future.

## Conclusion

So far, our evidence appears to attribute the main cause for accumulation of DCs mainly to a disrupted maturation process in fatal COVID-19 cases. Given the higher viral concentration in stage 1 lung lobes, these samples would be expected to at least hint an increased count of mDC expressing maturation markers in comparison to later stages. On the contrary–while the absolute count of CD86^+^ and CD11c^+^CD83^+^ cells needs to be viewed in the context of the overall larger mDC population in later stages, the CD11c^+^MHC II^+^ fractions directly indicate a near constant share of activated mDCs. In summary, dendritic cells failing to adequately prompt an adaptive immune reaction might be another component in the mismanaged immune response of severe COVID-cases. Given that SARS-CoV-2 is likely to remain an immense public health issue in the near future, immune evasion strategies need to be further examined.

## Data Availability Statement

The datasets presented in this study can be found in online repositories. The names of the repository/repositories and accession number(s) can be found at: https://figshare.com/articles/dataset/APC_phenotyping_and_cell_count_results/15384888.

## Ethics Statement

The studies involving human participants were reviewed and approved by Internal review board of the University Medical Center of Augsburg (BKF No. 2020–18) and the Ethics Committee of the University of Munich (Project Number 20–426, COVID-19 registry of the UKA). The patients/participants provided their written informed consent to participate in this study.

## Author Contributions

BM: conceptualization. AT and LB: image analysis. LB, BG, TS, BM, and KH: general analysis. RC, BM, and TS: funding acquisition. LB, BG, ÉS, RC, BM, and TS: investigation. BM, AT, and LB: methodology. BM and BG: administration. LB, BM, RC, KH, ÉS, OS, CR, and MW: writing, reviewing, and editing. All authors contributed to the article and approved the submitted version.

## Funding

This study received fundings amounting to €148,000 by the Bavarian Ministry of Science and the Arts.

## Conflict of Interest

The authors declare that the research was conducted in the absence of any commercial or financial relationships that could be construed as a potential conflict of interest.

## Publisher's Note

All claims expressed in this article are solely those of the authors and do not necessarily represent those of their affiliated organizations, or those of the publisher, the editors and the reviewers. Any product that may be evaluated in this article, or claim that may be made by its manufacturer, is not guaranteed or endorsed by the publisher.
