# Supplementary material for: Impaired Dendritic Cell Homing in COVID-19
Source: Front Med (Lausanne). 2021 Nov 4;8:761372. doi: 10.3389/fmed.2021.761372 (PMC8601231; doi:10.3389/fmed.2021.761372)
Supplement: Supplementary file 1 [file Data_Sheet_1.docx]

Supplementary Material

# Supplementary Figures and Tables

## Supplementary Tables

|  | **Upper right lobe** | **Middle lobe** | **Lower right lobe** | **Upper left lobe** | **Lower left lobe** |
| --- | --- | --- | --- | --- | --- |
| **Non-DAD** | 1 | 1 | 2 | 2 | 3 |
| **Stage 1** | 3 | 5 | 3 | 2 | 5 |
| **Stage 2** | 8 | 7 | 12 | 10 | 6 |
| **Stage 3** | 2 | 0 | 1 | 0 | 0 |
| **Sum** | 14 | 13 | 18 | 14 | 14 |

Supplementary Table 1. DAD-Stages found in the respective lobes of the samples analyzed. Stage 2 (organizing DAD) was the most prevalent stage in all lobes.

## Supplementary Figures


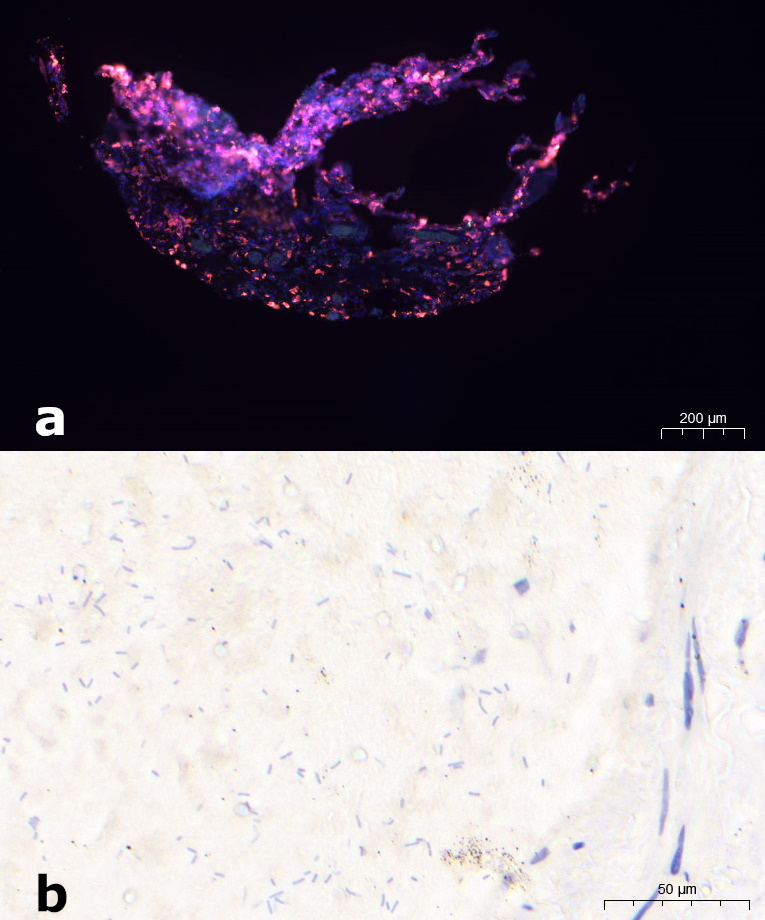


**Supplementary Figure 1.** a: Crumpled lung core. This core does not show an abnormal reaction to the stains but is of limited value as large parts of it cannot be assessed.

b: Example of a lung lobe that had to be discarded due to severe autolysis. CD8 stain, lower right lobe. The massive colonization of bacteria in this image is not depicting a bacterial superinfection but rather a post-mortem artifact, probably due to fixation issues.


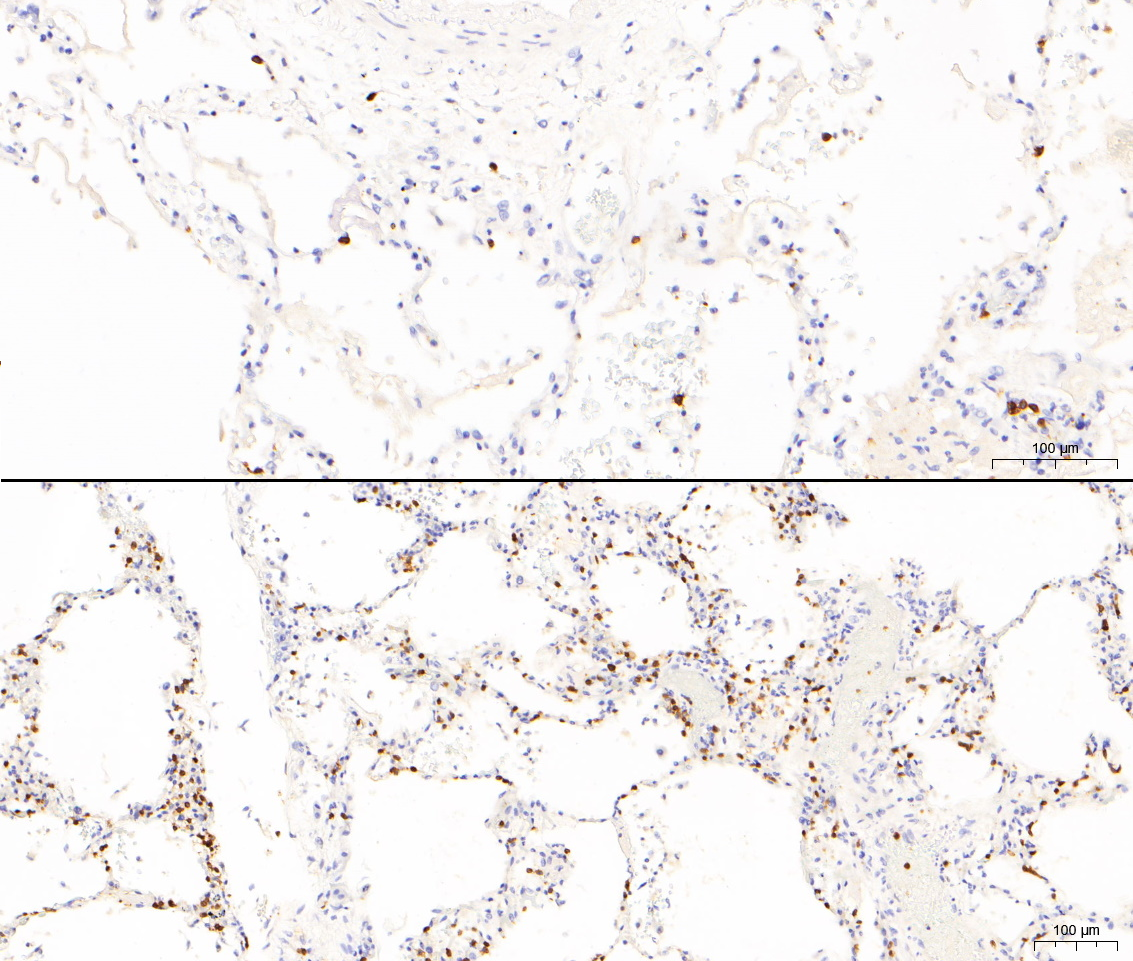


**Supplementary Figure 2.** Stage 2 DAD lower right lobe in conventional IHC. The upper image shows the rather weak CD8 reaction, while the lower image (CD3) confirms an abundance of t-cells.

**Supplementary Figure 3.** Simple linear regression of mean rt-qPCR value (ORF1-gene, N-gene, S-gene) and the fraction of MHC class II+ CD11c+ cells/all mDC. Data was available for 63 lung lobes. P = 0.6256.


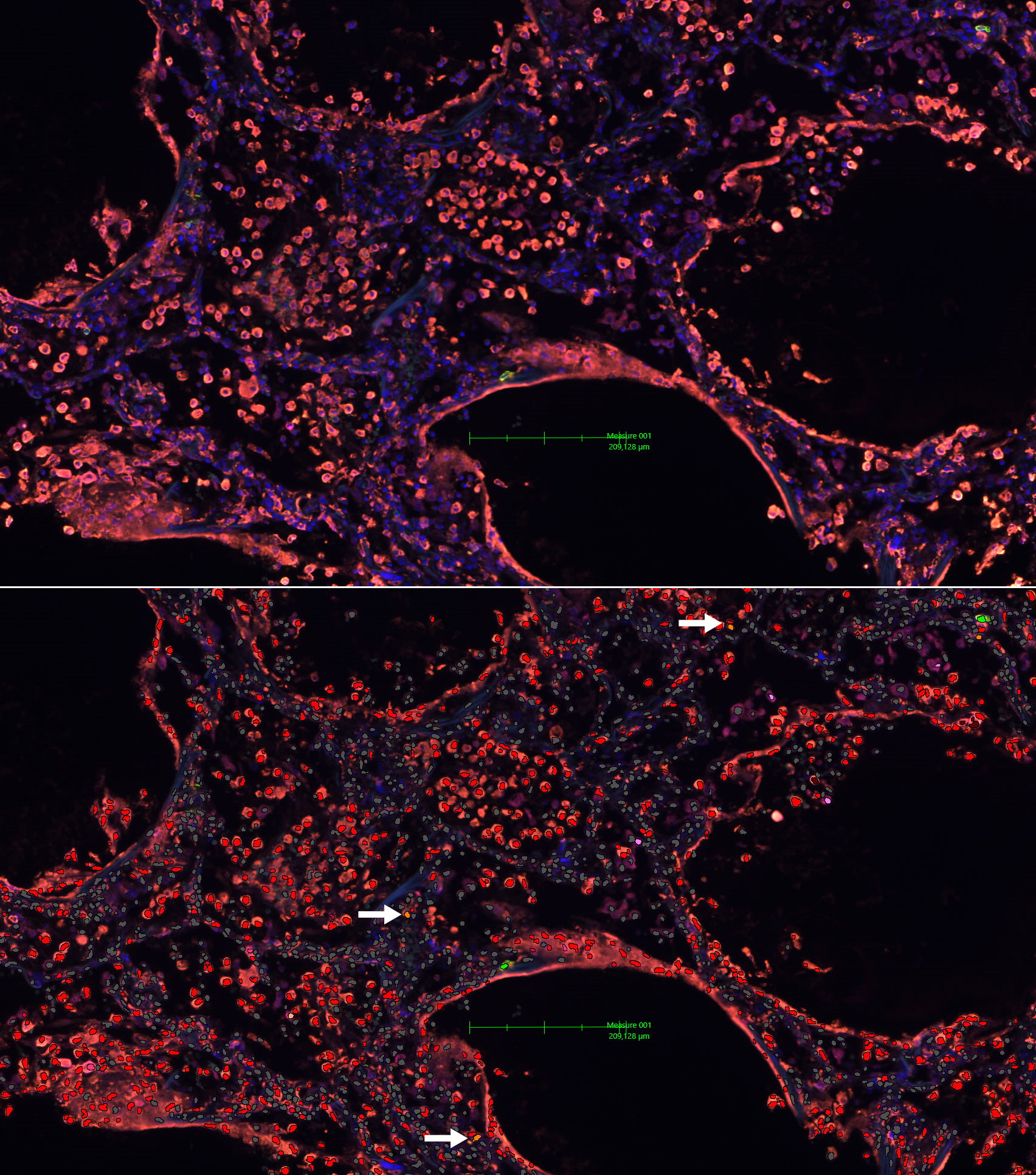


**Supplementary Figure 4.** Stage 1 DAD lung core. Massive infiltrate of alveolar macrophages (CD68/163 cocktail, light orange). The strong TRITC signal conceals the less prevalent Cy5 signal of the CD11c stain (bright orange). AI helps detect signal combinations that are indiscernible to the human eye. Bright green: CD20.


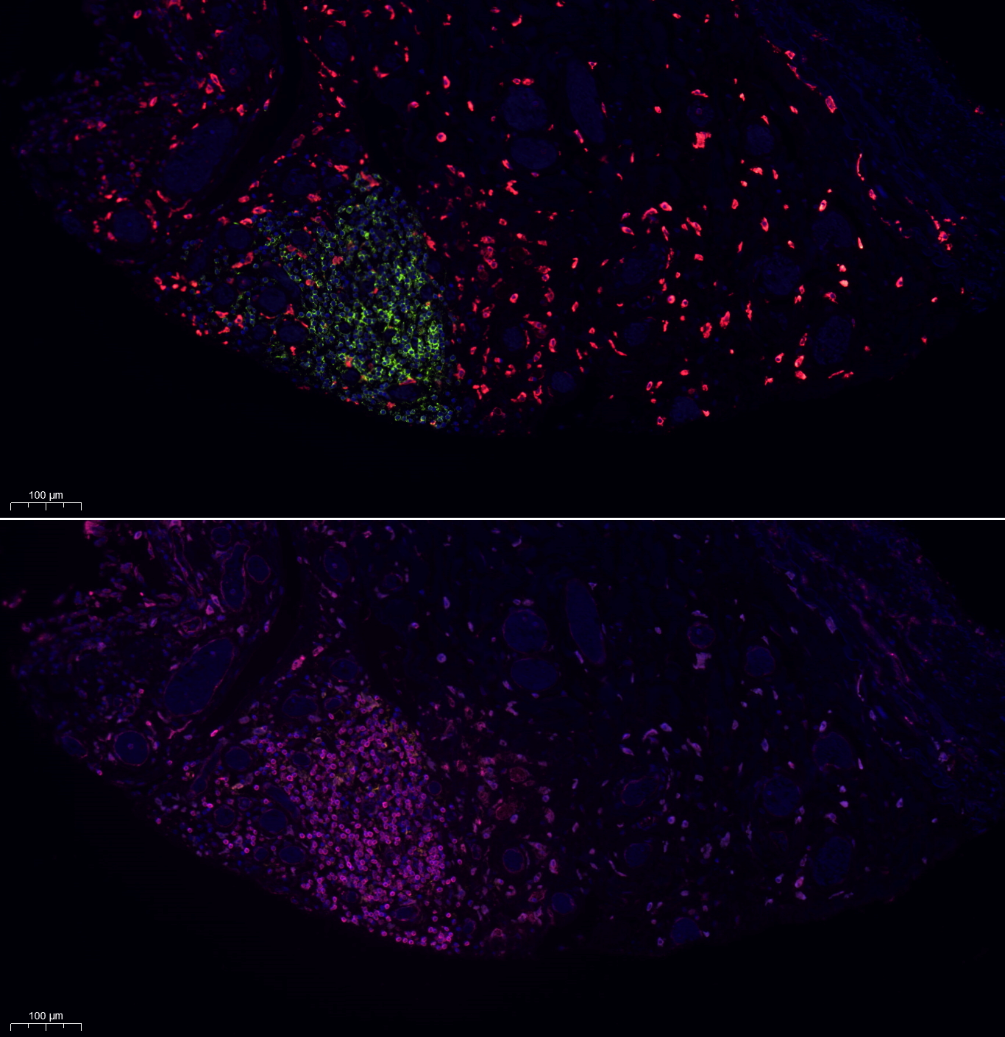


**Supplementary Figure 5.** Larger accumulation of CD20+ cells in a non-DAD lung core. FITC (bright green) is used to mark CD20, TRITC (deep pink) marks CD68/163, Cy5 (orange) marks CD11c. MHC class II is displayed by Cy7 (bright pink). For better visibility, the upper image displays CD20 and CD68/163, while the image below shows CD11c and MHC class II. DAPI (blue) is shown in both images). Screenshot created with the Case Viewer software by 3D Histech.

**Supplementary Figure 6.** Planned and actual sample size. Due to processing issues or autolysis not all retrieved lobes could be analyzed.


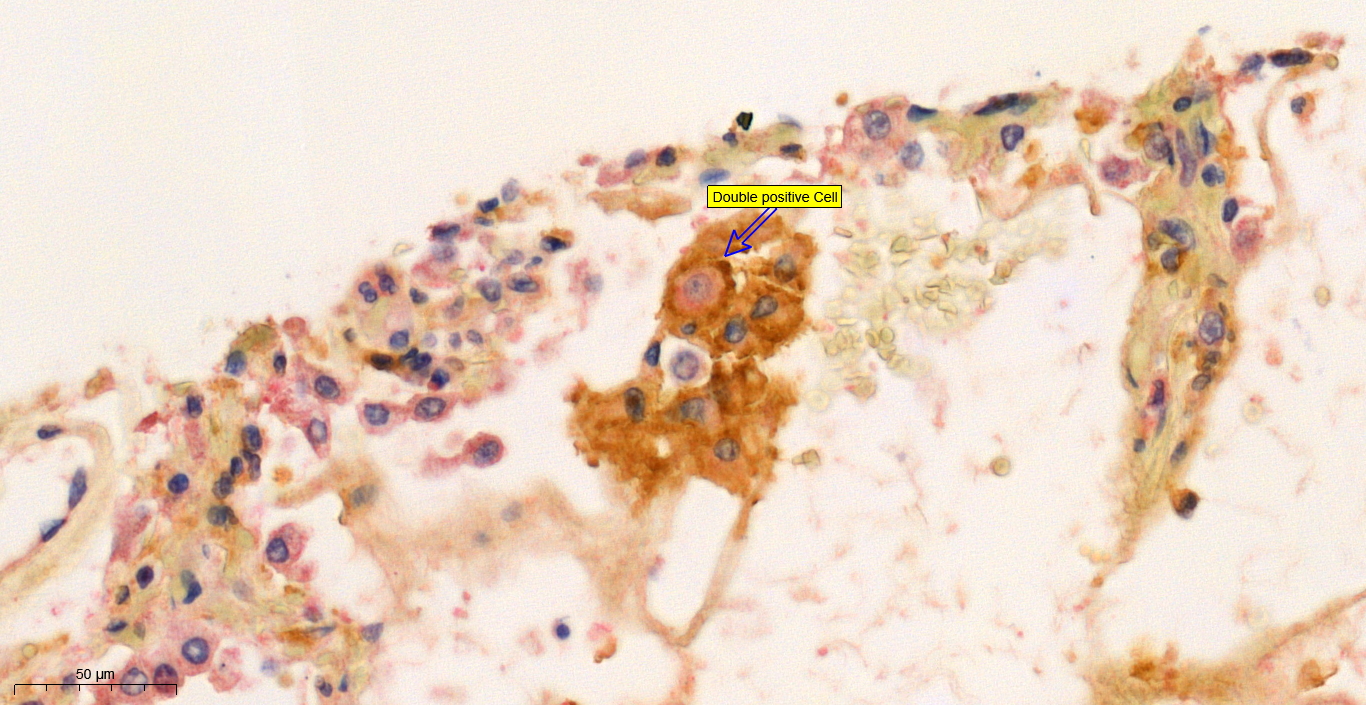


**Supplementary Figure 7.** Duplex staining of CD11c (DAB) and CD86 (fast red). The arrow marks a double positive cell that was relatively easy to discern.


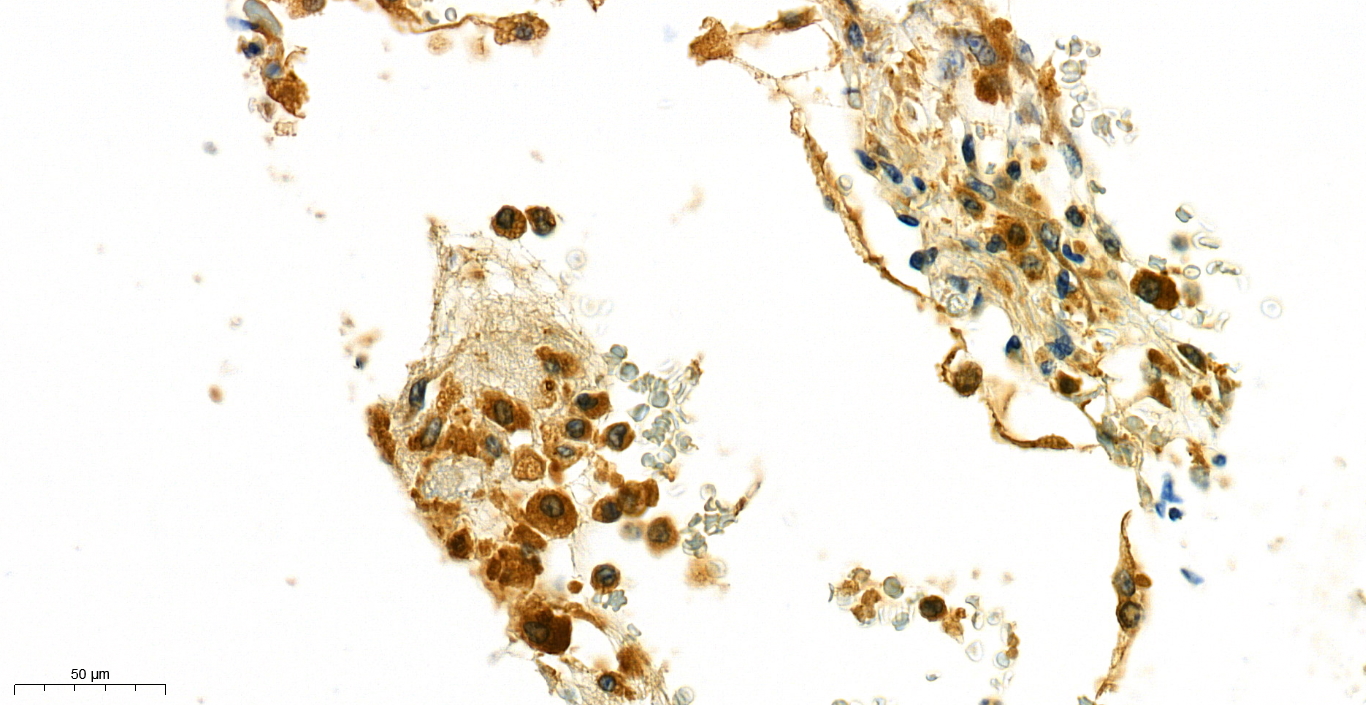


**Supplementary Figure 8a**. Unaltered scan of a CD80 staining of a stage 2 lung. CD80 is apparently expressed by a large number of cells, in varying intensity. Background staining further hampers the analysis.


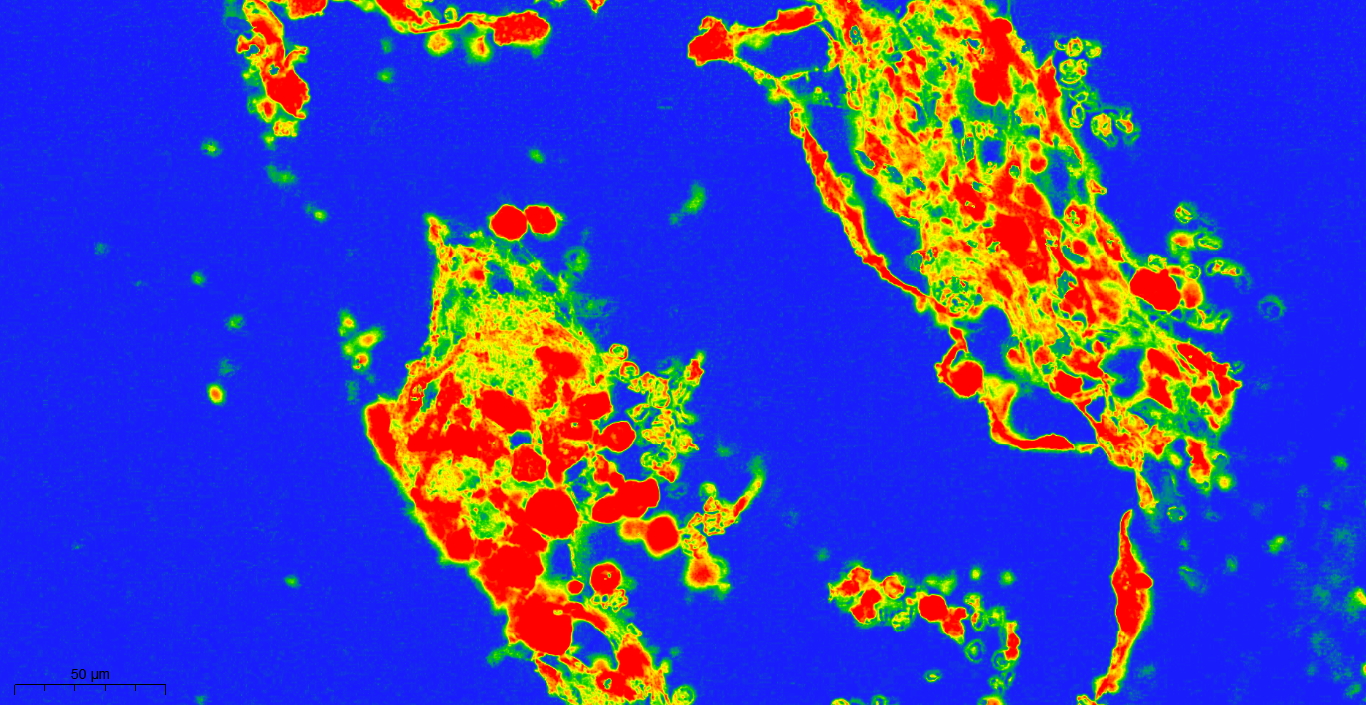


**Supplementary Figure 8b**. Heatmap of brown color signals of 8a. It is apparent that the heatmap in this unaltered picture is of little help in distinguishing positive and negative cells.


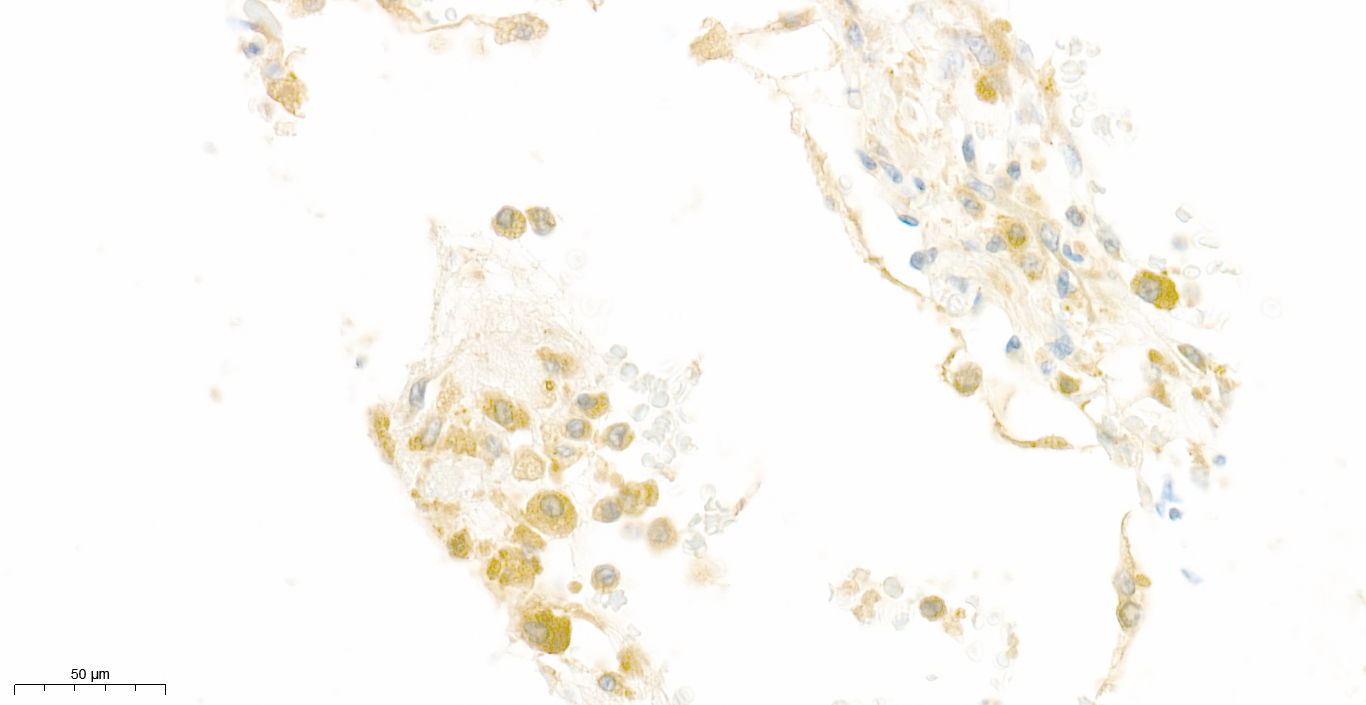


**Supplementary Figure 8c**. Raising the γ-value of the image to 1.8 helps to differentiate strong signals and weaker background signals.


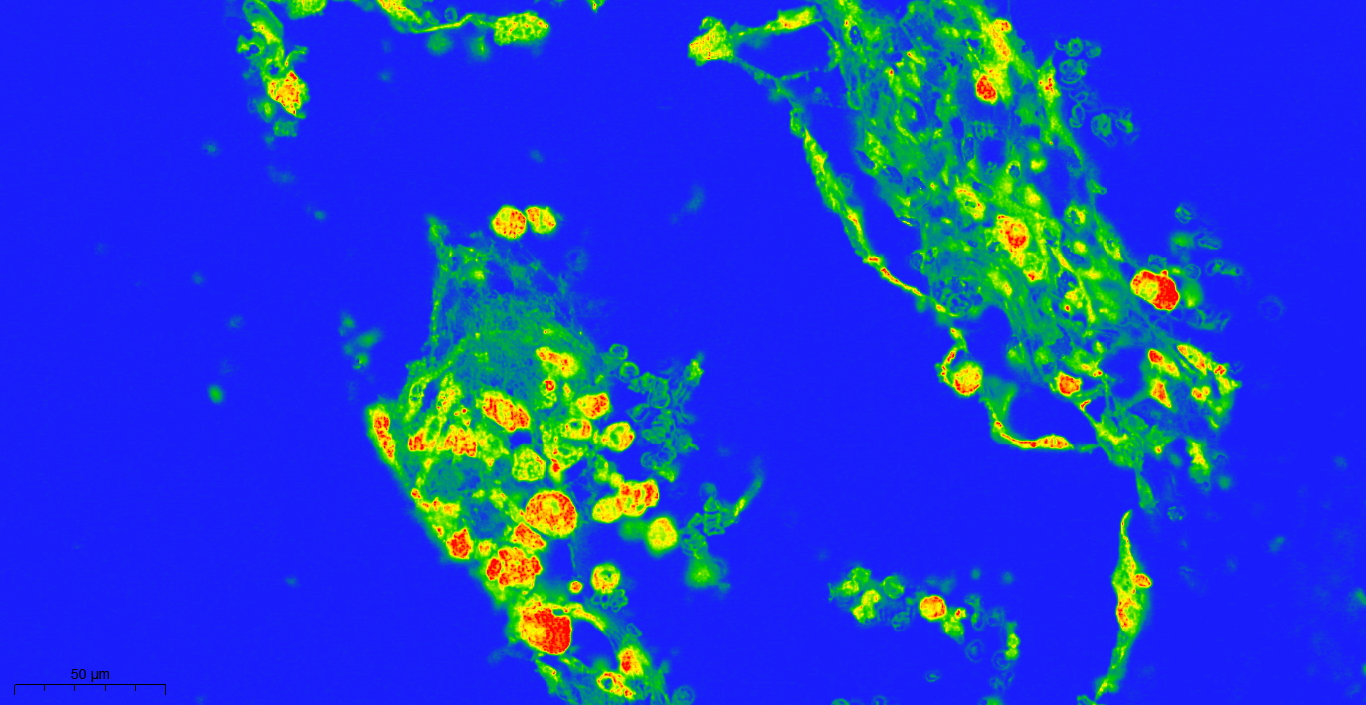


**Supplementary Figure 8d**. Heatmap of the same area as 8b, but with altered gamma-value. Positive and negative cells can be easily distinguished and could possibly help achieving a better interrater-reliability. All CD80 stained images were analyzed with the same γ-value.

**
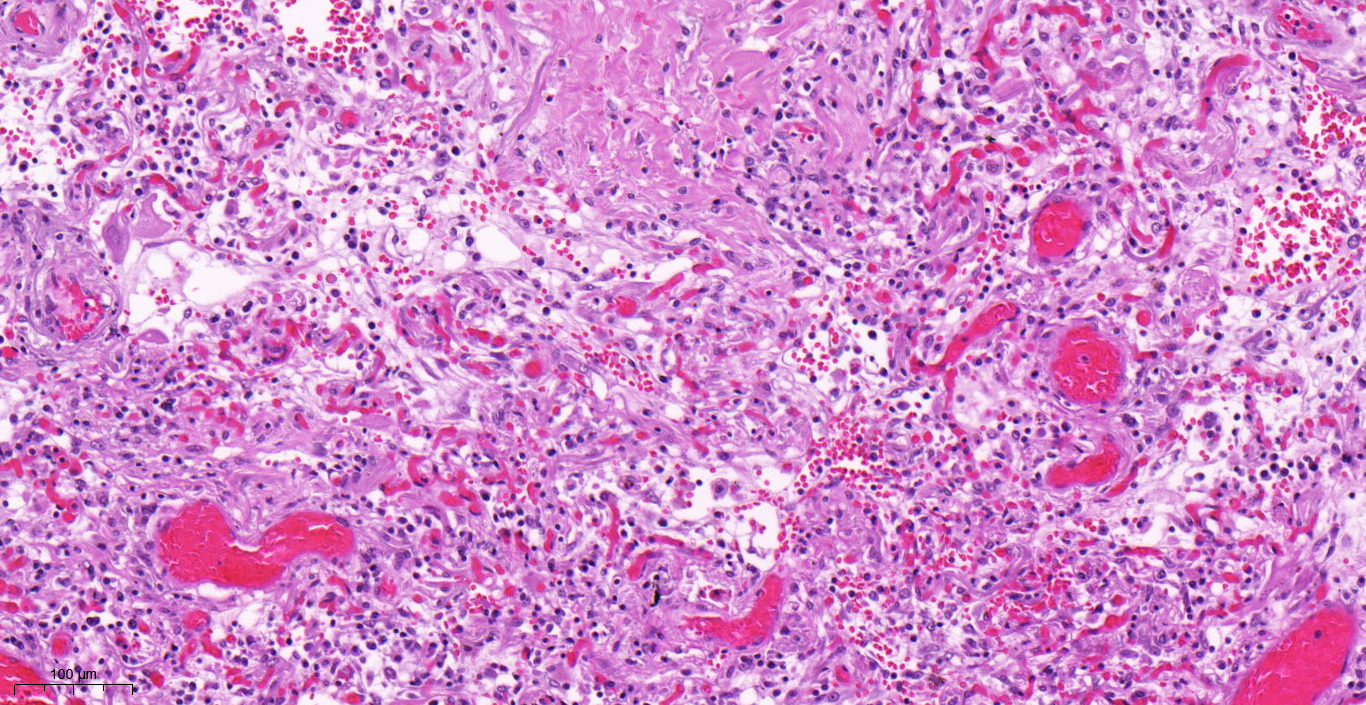
**

**Supplementary Figure 9**. Example of a stage 2 (proliferative/organizing DAD) lung lobe at low power.
